# The Effectiveness and Safety of Direct Oral Anticoagulants in Obese Patients With Atrial Fibrillation: A Network Meta-Analysis

**DOI:** 10.7759/cureus.41619

**Published:** 2023-07-10

**Authors:** Qais M Salah, Sagar Bhandari, Ali Chand, Saif Khan, Syed Haider Ali Tirmzi, Majed Sheikh, Khaldoun Khreis, Sujith K Palleti

**Affiliations:** 1 Internal Medicine, Al-Quds University Faculty of Medicine, Jerusalem, PSE; 2 Cardiology, Tucson Medical Center, Tucson, USA; 3 Medicine, Shaikh Khalifa Bin Zayed Medical and Dental College, Lahore, PAK; 4 Medicine, North Manchester Hospital, Manchester, GBR; 5 Medicine, Northern Care Alliance, Royal Oldham Hospital, Manchester, GBR; 6 Cardiology, Royal Free London NHS Foundation Trust, London, GBR; 7 Pediatrics, University of Pécs Medical School, Pécs, HUN; 8 Nephrology, Edward Hines Jr. Veterans Administration Hospital, Hines, USA; 9 Nephrology, Loyola University Medical Center, Maywood, USA

**Keywords:** meta-analysis, obesity, atrial fibrillation, warfarin, direct oral anticoagulants

## Abstract

Atrial fibrillation (AF) is a cardiac condition characterized by an irregular heart rhythm, which is increasingly prevalent in the modern era. All international guidelines strongly advise the administration of anticoagulants to individuals with AF who are at high risk of stroke. These guidelines recommend the use of direct oral anticoagulants (DOACs) over warfarin because warfarin is significantly associated with increased rates of major bleeding, numerous interactions with food and drugs, and the necessity for frequent monitoring. The aim of this study is to compare the effectiveness and safety of direct oral anticoagulants (DOACs) in obese patients with atrial fibrillation. Two authors independently conducted a comprehensive literature search using electronic databases including PubMed, CINAHL, and EMBASE from inception to June 1, 2023. The efficacy outcome assessed in this meta-analysis included the composite of stroke and systemic embolism. For safety analysis, major bleeding events were compared among the study groups. Eleven studies fulfilled all the inclusion criteria and were included in the present meta-analysis enrolling 144,502 patients. In this study, DOACs demonstrate superior efficacy in preventing stroke/systemic embolism compared to warfarin. Among the DOACs, apixaban emerged as the most effective, followed by rivaroxaban, warfarin, and dabigatran. In terms of safety, apixaban was also found to be the most favorable treatment option, followed by rivaroxaban, dabigatran, and warfarin. In summary, our study concludes that apixaban exhibited greater effectiveness and safety when compared to other DOACs and warfarin in obese patients with AF.

## Introduction and background

Atrial fibrillation (AF) is a cardiac condition characterized by an irregular heart rhythm, which is increasingly prevalent in the modern era. AF can stem from various factors, including valvular heart diseases and non-valvular causes such as thyroid issues, hypertension, sleep apnea, exposure to substances that stimulate the heart, stress, or other unknown reasons. Research indicates that many AF patients do not have underlying valvular heart disease [1]. Non-valvular AF can lead to complications such as blood clot formation, resulting in stroke and systemic embolism [2]. Typically, patients with non-valvular AF require anticoagulant medication for proper management [3]. In 2017-2018, the estimated prevalence of adult obesity in the United States was 42%, and it is expected to potentially reach 50% by 2030 [4]. Apart from various health issues, obesity is linked to an increased likelihood of developing non-valvular atrial fibrillation and thrombotic events [5]. In obese individuals who develop non-valvular atrial fibrillation, the condition tends to be more severe and persistent [6]. Obesity is one of the important risk factors for AF due to its underlying mechanisms that physiologically affect AF. For instance, obesity is often associated with metabolic disturbances such as insulin resistance, hyperlipidemia, and hypertension. These metabolic factors can promote the development of AF by affecting atrial substrate and function [7]. Furthermore, obesity leads to increased blood volume and cardiac output, resulting in chronic hemodynamic alterations. These changes can cause left atrial enlargement and stretch, leading to atrial remodeling and increased susceptibility to AF. Approximately one out of every five cases of AF is associated with obesity, to the point that each incremental rise in body mass index (BMI) corresponds to a 4%-5% increase in the risk of developing AF [8].

In general, patients without clinical stroke risk factors do not need antithrombotic therapy, while patients with stroke risk factors (i.e., CHA2DS2-VASc score of 1 or more for males and 2 or more for females) are likely to benefit from oral anticoagulants (OACs). All international guidelines strongly advise the administration of anticoagulants to individuals with AF who are at high risk of stroke (CHA2DS2-VASc score of 1 or more for males and 2 or more for females) [9]. These guidelines recommend the use of direct oral anticoagulants (DOACs) over warfarin because warfarin is significantly associated with increased rates of major bleeding, numerous interactions with food and drugs, and the necessity for frequent monitoring [10,11]. The modified pathophysiology observed in obese adults can impact the pharmacological effects of anticoagulants such as warfarin, necessitating a higher dosage and a lengthier period to achieve therapeutic targets in comparison to individuals with normal weight [12]. This circumstance may contribute to unfavorable outcomes such as stroke and hospitalization resulting from inadequate dosage of anticoagulant medication.

Numerous systematic reviews have focused on DOACs and their application in obesity, attracting significant interest [13,14]. However, the recommendations from these studies seem to present conflicting findings. The impact of the obesity paradox concerning AF, as well as comprehensive data comparing the effectiveness of DOACs to warfarin, remains uncertain. For instance, only a small percentage (1.4%) of participants in the Apixaban for Reduction In Stroke and Other ThromboemboLic Events in Atrial Fibrillation (ARISTOTLE) trial [15] weighed over 140 kg, indicating an underrepresentation of this population. Concerns regarding the use of DOACs in severely obese adults (with a BMI of 40 kg/m^2^ or higher) have been raised by both the International Society on Thrombosis and Haemostasis (ISTH) and the European Society of Cardiology (ESC) Working Group on Thrombosis. This is primarily due to the lack of sufficient clinical data or its absence [16]. The ISTH has suggested that DOACs should not be used in individuals with a BMI exceeding 40 kg/m^2^ or weighing over 120 kg [17]. Currently, no randomized controlled trials (RCTs) of DOACs administered specifically to morbidly obese patients exist. Furthermore, there is a lack of studies comparing different oral anticoagulants in obese patients with AF. Therefore, in this study, we sought to compare different DOACs with warfarin and with each other in obese patients with AF using meta-analysis. The aim of this meta-analysis is to compare the effectiveness and safety of DOACs in obese patients with atrial fibrillation using network meta-analysis. We used network meta-analysis as it allows for the simultaneous comparison of multiple treatments (apixaban, dabigatran, rivaroxaban, and warfarin) within the same analysis. This approach enables a comprehensive assessment of the relative effectiveness and safety of these different anticoagulants in obese patients with atrial fibrillation.

## Review

Methodology

This meta-analysis was conducted following the Preferred Reporting Items for Systematic Reviews and Meta-Analyses of Network Meta-Analyses (PRISMA-NMA) guidelines.

Search Strategy

Two authors independently conducted a comprehensive literature search using electronic databases including PubMed, CINAHL, and EMBASE from inception to June 1, 2023. The search used keywords such as "atrial fibrillation," "obese," "direct oral anticoagulants," and "warfarin," along with their synonyms and relevant medical subject heading (MeSH) terms. All retrieved records were imported into EndNote X9, duplicates were removed, and two reviewers screened the articles based on their titles and abstracts. The full texts of all potentially eligible records were obtained, and a detailed assessment was performed based on predefined inclusion and exclusion criteria. Additionally, the reference lists of included studies were manually searched.

Inclusion and Exclusion Criteria

The following criteria were used for study inclusion: (1) randomized controlled trials (RCTs) or observational cohorts (prospective or retrospective); (2) patients with obesity (defined by BMI (>30 kg/m^2^) or ICD-9 or 10 codes) and atrial fibrillation, aged 18 years or older, who received any of the three DOACs (apixaban, rivaroxaban, and dabigatran) or warfarin; and (3) reporting of any efficacy or safety outcomes. Studies that included patients other than those with atrial fibrillation were excluded. Reviews, editorials, and case reports were also excluded. We excluded studies published in languages other than English.

Data Extraction, Outcomes, and Quality Assessment

Two authors independently extracted data from each of the included studies. The extracted data included authors' names, publication year, study design, medications used, and patient number. They also extracted data on the methodological quality of the studies, baseline characteristics of participants, duration of follow-up, and number of recorded events. In case of any disagreements between the two authors during the data extraction process, they engaged in thorough discussions to reach a consensus, with the corresponding author making the final decision. The efficacy outcome assessed in this meta-analysis included the composite efficacy outcomes (including stroke and systemic embolism). For safety analysis, major bleeding events were compared among the study groups.

Two authors independently assessed the quality of the studies. The quality of these studies was assessed using the Newcastle-Ottawa Scale (NCOS) because each of the study that was included in this meta-analysis was a cohort. It evaluates three key areas: the selection of study groups, the comparability of groups, and the ascertainment of either the exposure or outcome of interest.

Statistical Analysis

The network meta-analysis was performed using Stata software version 16.0 (StataCorp LLC, College Station, TX, USA). A network diagram was drawn. Each node in the diagram represented a specific intervention, while the size of the node reflected the sample size, and the thickness of the lines represented the number of studies comparing each pair of treatments. For the network meta-analysis, a Bayesian approach was employed. Pairwise comparisons were done using odds ratio (OR) with 95% confidence interval (CI). Surface under the cumulative ranking (SUCRA) score was used to rank the intervention. It is a statistical measure used in meta-analysis to rank different treatment interventions based on their probabilities of being the best treatment for a specific outcome. It provides a summary measure of the relative treatment efficacy or effectiveness across multiple interventions. Heterogeneity was assessed using I-square statistics. An I-square value of more than 50% was considered significant for heterogeneity.

Results

**Figure 1:**
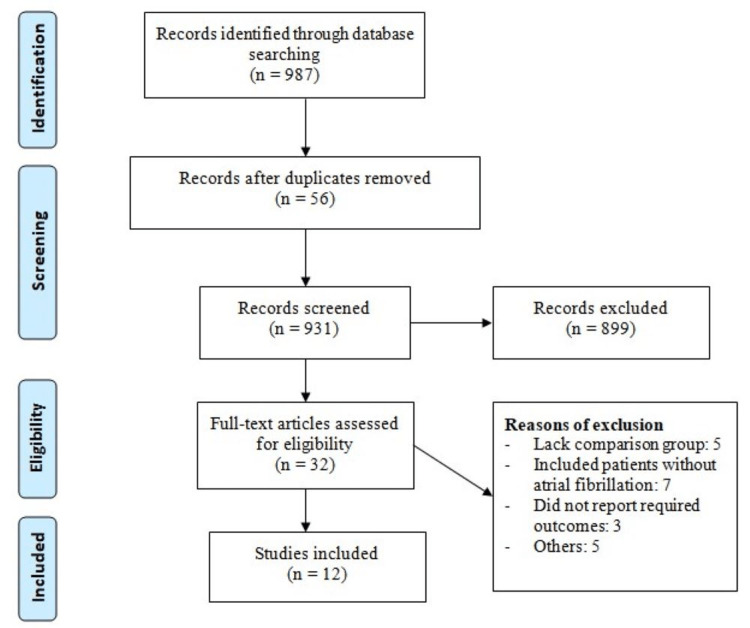
PRISMA flowchart of study selection PRISMA: Preferred Reporting Items for Systematic Reviews and Meta-Analyses

A total of 987 records were obtained from online database searching. After removing 56 duplicate articles, 931 articles were left for title and abstract screening. A total of 899 studies were excluded based on title and abstracts. A total of 32 studies were included for full-text screening. Eleven studies fulfilled all the inclusion criteria and were included in the present meta-analysis enrolling 144,502 patients. Figure 1 shows the study selection process. Table 1 gives the characteristics of all the included studies. Table 2 shows the quality assessment of the included studies.

**Table 1:** Characteristics of the included studies NR: not reported, BMI: body mass index

Author name	Year	Region	Study design	Groups	Sample size	Follow-up	BMI	Age (years)	Males (%)
Alberts et al. [18]	2022	United States	Retrospective cohort	Rivaroxaban	21,574	25 months	BMI ≥ 30 kg/m^2^	65.1/65.3	64.1/63.9
				Warfarin	21,574				
Berger et al. [19]	2021	United States	Retrospective cohort	Rivaroxaban	10,555	36 months	BMI ≥ 30 kg/m^2^	58.5/60.9	69.5/67.6
				Warfarin	5,080				
Boivin-Proulx et al. [20]	2022	Canada	Retrospective cohort	Apixaban	539	12 months	Used ICD-9 and ICD-10 codes	74.22/71.91/72.83	44.45/45.57/43.68
				Rivaroxaban	403				
				Warfarin	1,253				
Briasoulis et al. [21]	2021	United States	Retrospective cohort	Apixaban	4,471	24 months	BMI ≥ 40 kg/m^2^	67.3/67.2/67.3/67.2	92/90/93/89
				Rivaroxaban	3,299				
				Dabigatran	3,246				
				Warfarin	13,417				
Chugh et al. [22]	2023	United States	Retrospective cohort	Apixaban	155	12.5 months	BMI ≥ 30 kg/m^2^	NR	NR
				Rivaroxaban	335				
				Dabigatran	393				
Costa et al. [23]	2020	United States	Retrospective cohort	Rivaroxaban	1,969	27.6 months	BMI ≥ 30 kg/m^2^	NR	20.7/49.7
				Warfarin	1,969				
Deitelzweig et al. [24]	2021	Canada	Retrospective cohort	Apixaban	13,604	8 months	BMI ≥ 30 kg/m^2^	72.3/72.3	51.3/51.6
				Warfarin	12,918				
Kido et al. [25]	2019	United States	Retrospective cohort	Apixaban	19	45 months	BMI ≥ 40 kg/m^2^	NR	NR
				Rivaroxaban	25				
				Dabigatran	20				
				Warfarin	64				
Kushnir et al. [26]	2019	United States	Retrospective cohort	Apixaban	103	10 months	BMI ≥ 30 kg/m^2^	65.9/60.9/66.8	44/45/41
				Rivaroxaban	174				
				Warfarin	152				
Perales et al. [27]	2020	United States	Retrospective cohort	Rivaroxaban	37	12 months	BMI ≥ 40 kg/m^2^	56.0/55.0	48.0/45.0
				Warfarin	30				
Peterson et al. [28]	2019	United States	Retrospective cohort	Rivaroxaban	3,563	10 months	BMI ≥ 30 kg/m^2^	62.9/62.9	53.9/54.0
				Warfarin	3,563				
Weir et al. [29]	2021	United States	Retrospective cohort	Rivaroxaban	9,999	28 months	BMI ≥ 30 kg/m^2^	70.0/70.2	58.8/58.0
				Warfarin	9,999				

**Table 2:** Quality assessment of the included studies

Author name	Selection	Comparison	Outcome	Overall
Alberts et al. [18]	3	2	3	Good
Berger et al. [19]	3	1	2	Fair
Boivin-Proulx et al. [20]	4	1	2	Good
Briasoulis et al. [21]	3	2	3	Good
Chugh et al. [22]	4	2	2	Good
Costa et al. [23]	4	2	2	Good
Deitelzweig et al. [24]	2	1	2	Fair
Kido et al. [25]	3	2	3	Good
Kushnir et al. [26]	4	2	3	Good
Perales et al. [27]	3	2	3	Good
Peterson et al. [28]	3	2	2	Good
Weir et al. [29]	4	2	3	Good

Network Meta-Analysis Results

**Figure 2:**
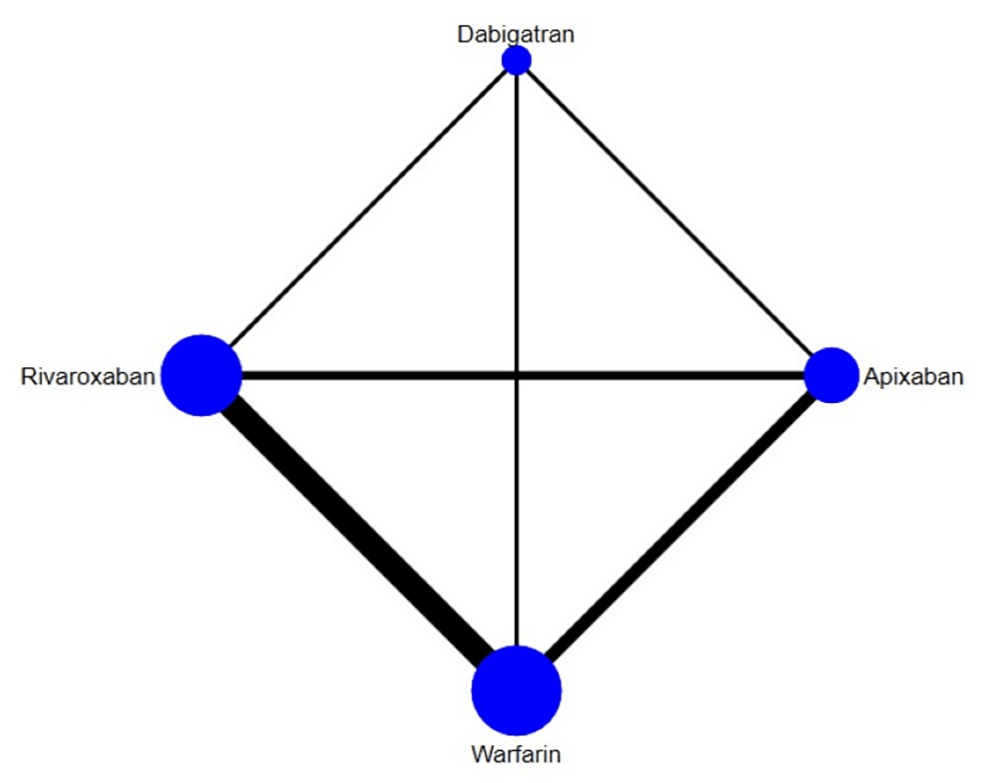
Network plot (composite outcome)

**Figure 3:**
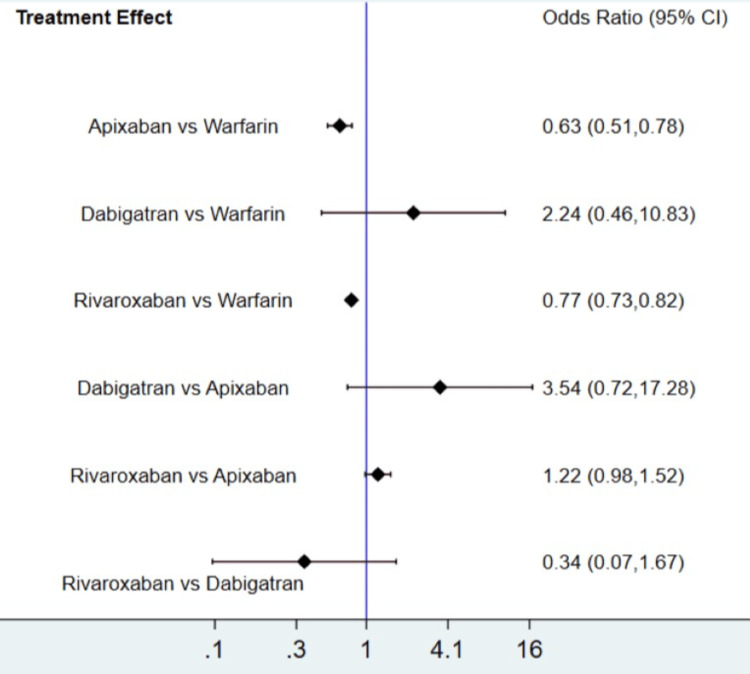
Treatment effect (composite outcome) CI: confidence interval

The results for the primary efficacy outcomes of stroke or systemic embolism are shown in Figure 2 and Figure 3. Overall, the risk of composite efficacy outcome was lower in apixaban and rivaroxaban compared to warfarin. However, dabigatran was non-inferior to warfarin in terms of prevention of composite efficacy outcome. In the prophylaxis of stroke or systemic embolism, apixaban was the best among the other two DOACs (rivaroxaban and dabigatran) and warfarin as shown in Table 3. The SUCRA score of each drug showed that in the prevention of composite efficacy outcome, apixaban ranked the best, followed by rivaroxaban, warfarin, and dabigatran. The heterogeneity was analyzed, and the results showed that the heterogeneity of the study was low (18%). The results of Egger's tests showed no obvious publication bias in the efficacy outcome (p-value: 0.22).

**Table 3:** SUCRA score SUCRA: surface under the cumulative ranking

Intervention	Composite outcome	Stroke	Major bleeding
Apixaban	1	0.8	1
Rivaroxaban	0.6	0.6	0.5
Dabigatran	0.1	0.3	0.4
Warfarin	0.3	0.1	0.1

**Figure 4:**
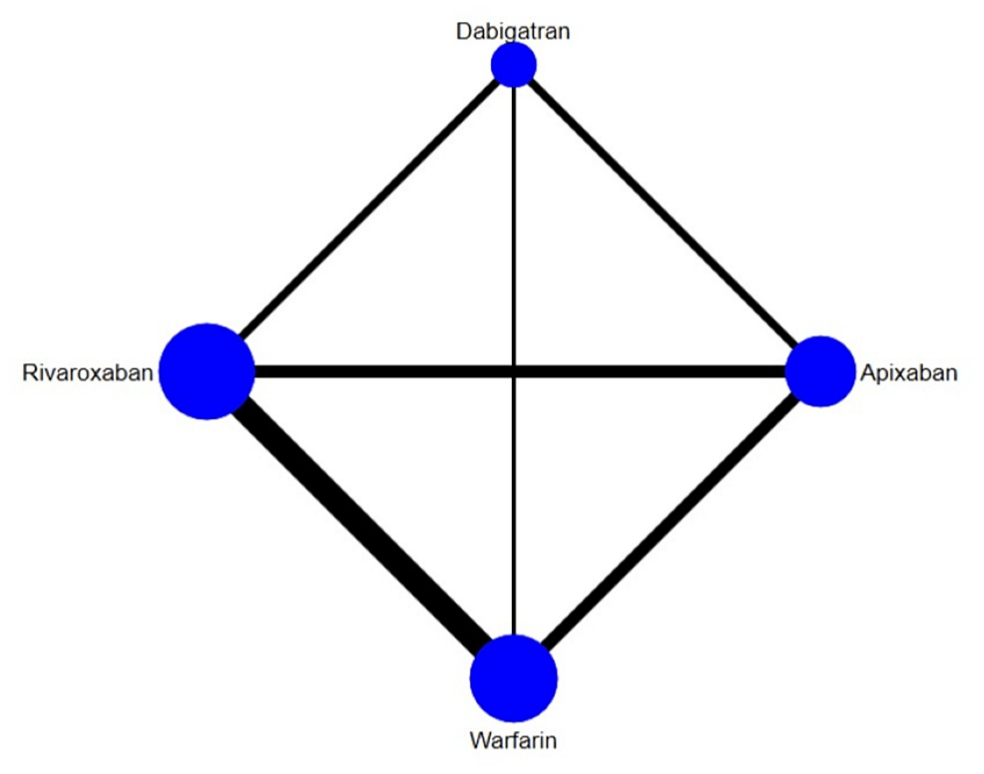
Network plot (stroke)

**Figure 5:**
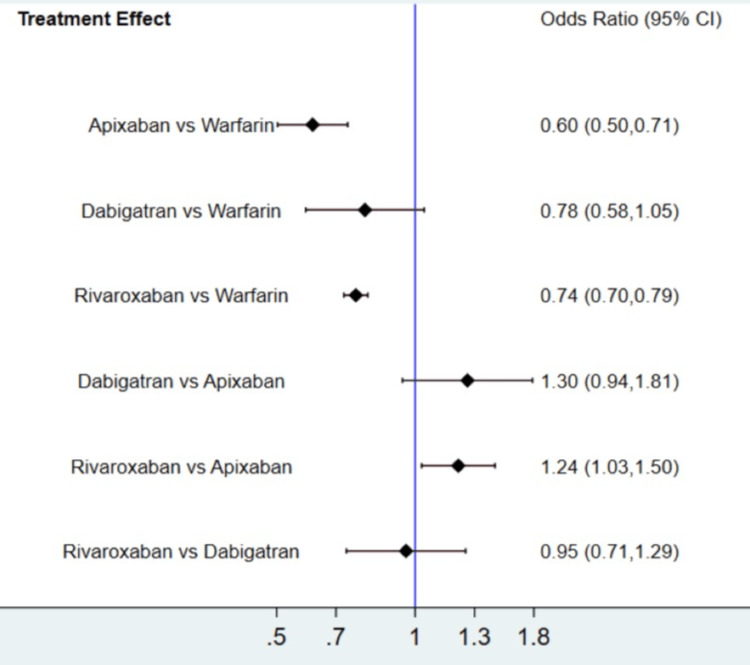
Treatment effect (stroke) CI: confidence interval

The results for the comparison of stroke among interventions are shown in Figure 4 and Figure 5. Overall, the risk of stroke was lower in apixaban and rivaroxaban compared to warfarin. However, dabigatran was non-inferior to warfarin in terms of prevention of stroke. The SUCRA score of each drug showed that in the prevention of stroke, apixaban ranked the best, followed by rivaroxaban, dabigatran, and warfarin. The heterogeneity was analyzed, and the results showed that the heterogeneity of the study was low (32%). The results of Egger's tests showed no obvious publication bias in the efficacy outcome (p-value: 0.55).

**Figure 6:**
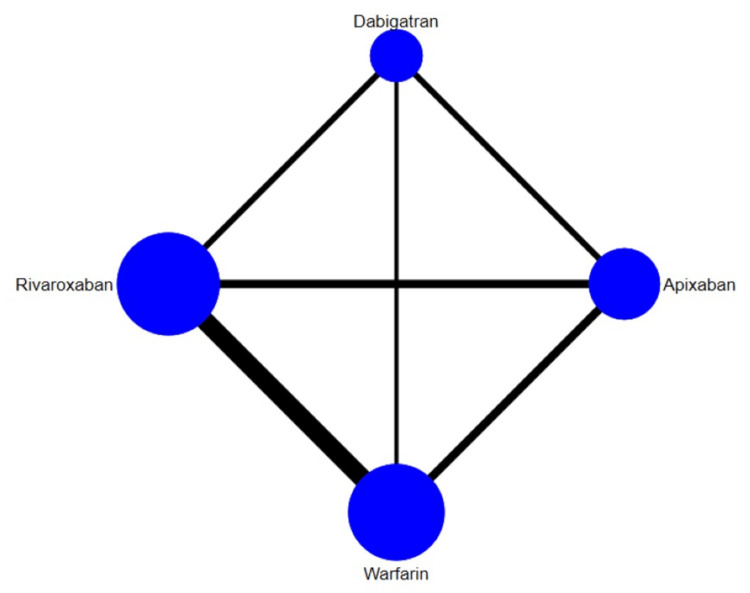
Network map (major bleeding events)

**Figure 7:**
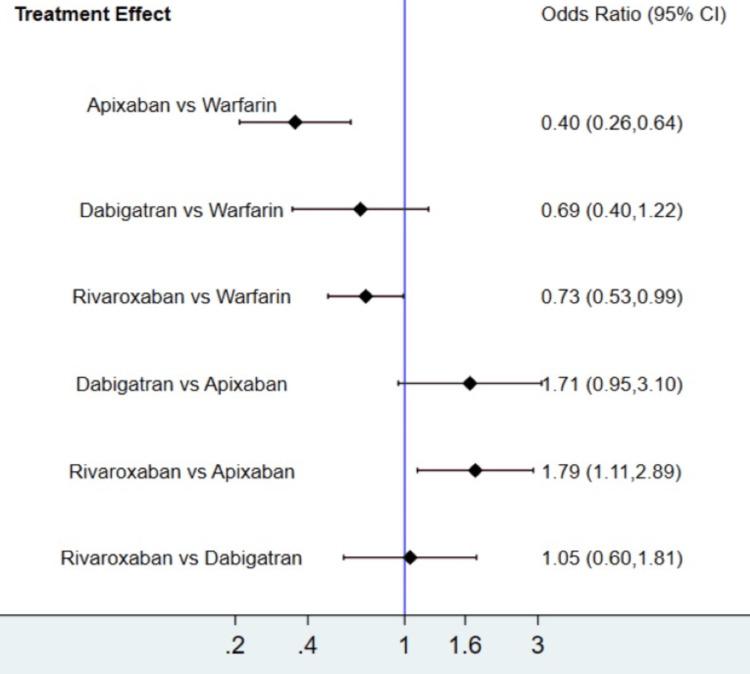
Treatment effect (major bleeding events) CI: confidence interval

The results for the major bleeding events are shown in Figure 6 and Figure 7. Overall, the risk of major bleeding events was lower in apixaban and rivaroxaban compared to warfarin. The risk of major bleeding events in patients receiving dabigatran was lower than in patients receiving warfarin, but the difference was statistically insignificant. The SUCRA score in Table 3 shows that apixaban was the best among all anticoagulants, followed by rivaroxaban, dabigatran, and warfarin. The heterogeneity was analyzed, and the results showed that the heterogeneity of the study was low (0%). The results of Egger's tests showed no obvious publication bias in the major bleeding outcome (p-value: 0.14).

Discussion

The aim of this network meta-analysis was to identify the most suitable medication for preventing stroke/systemic embolism in obese patients with AF. In this study, DOACs demonstrate superior efficacy in preventing stroke/systemic embolism compared to warfarin. Among the DOACs, apixaban emerged as the most effective, followed by rivaroxaban, warfarin, and dabigatran. In terms of safety, apixaban was also found to be the most favorable treatment option, followed by rivaroxaban, dabigatran, and warfarin. In summary, our study concludes that apixaban exhibited greater effectiveness and safety when compared to other DOACs and warfarin in obese patients with AF.

DOACs represent a substantial advancement and address various limitations associated with warfarin treatment. These DOACs offer several advantages, such as a rapid onset of action, convenient dosing, absence of routine therapeutic monitoring, and reduced interactions with other drugs and dietary factors. These factors contribute to an improved quality of life for both patients and their caregivers [30,31]. While DOACs have a more predictable pharmacokinetic profile compared to warfarin, it is important to note that the absence of dosage adjustment may pose a risk of under- or over-anticoagulation in patients with extreme body weights due to inconsistent bioavailability. However, our study presents reassuring findings that support the continued preference for DOACs due to their convenience.

A meta-analysis published in 2021, comparing DOAC with warfarin, including 89,494 morbidly obese patients with atrial fibrillation, showed DOACs to be safe and effective with statistical superiority in these patients, supporting the results of this present meta-analysis [32]. Another meta-analysis comparing the efficacy and safety of apixaban and rivaroxaban in patients with high BMI showed positive outcomes [33]. However, the meta-analysis did not include patients with AF.

The International Society on Thrombosis and Haemostasis (ISTH) expressed reservations in 2016 regarding the use of DOACs in obese patients, citing insufficient research on this specific population [17]. However, with the emergence of additional studies focusing on the utilization of apixaban and other DOACs in obese patients in recent years, it has been demonstrated that these medications offer potential advantages over warfarin in this patient group. As a result, the recommendation for their use in obese patients has been established. Apixaban and rivaroxaban had a similar positive response. However, the use of dabigatran in obese patients with AF required further confirmatory studies as the number of studies assessing the efficacy and safety of dabigatran in these patients is limited.

The present meta-analysis examined the efficacy and safety of direct-acting oral anticoagulants (DOACs) compared to warfarin in terms of major bleeding events. Among the DOACs, apixaban and rivaroxaban have shown superior effectiveness in reducing major bleeding events when compared to warfarin. Obese patients pose unique challenges when it comes to anticoagulant therapy due to altered pharmacokinetics and potential variations in drug efficacy and safety [16]. The physiological basis for the effectiveness of apixaban and rivaroxaban in reducing major bleeding events in obese patients lies in their pharmacological properties. Both apixaban and rivaroxaban are factor Xa inhibitors that exert their anticoagulant effects by selectively inhibiting factor Xa, thereby interrupting the coagulation cascade [34]. In obese individuals, alterations in the volume of distribution and increased body weight can influence drug pharmacokinetics, potentially leading to variations in drug exposure and response. However, apixaban and rivaroxaban have predictable pharmacokinetic profiles that allow for consistent anticoagulation effects, irrespective of body weight or BMI [35]. This predictability ensures that obese patients receive appropriate and effective anticoagulant therapy, reducing the risk of major bleeding events.

Study Limitations

There are several limitations to consider in our meta-analysis. Firstly, all the studies included in our analysis were observational in nature, which may introduce inherent biases and confounding factors that could impact the validity of our findings. Secondly, our analysis was limited by the availability of data on certain important outcomes, such as myocardial infarction and all-cause mortality. These endpoints were not adequately reported in the included studies, which reduces the comprehensiveness of our analysis. Furthermore, the intensity of obesity, as measured by variables such as body mass index (BMI), was not taken into consideration in our analysis due to the unavailability of data on patients' levels. This omission prevents us from assessing the impact of obesity severity on the outcomes of interest. Additionally, the inclusion criteria of studies regarding BMI varies from study to study as some studies used 30 kg/m^2^ and some used 40 kg/m^2^ to identify obese patients. Lastly, we encountered a limitation related to the number of studies assessing dabigatran, one of the DOACs included in our analysis. Therefore, in order to evident these findings, more studies were needed in obese patients with atrial fibrillation.

## Conclusions

In conclusion, our network meta-analysis of 12 studies suggests that direct-acting oral anticoagulants (DOACs) demonstrate superior efficacy and safety compared to warfarin in preventing stroke/systemic embolism in obese patients with atrial fibrillation (AF). Among the DOACs, apixaban emerged as the most effective and safest treatment option. These findings support the preference for DOACs over warfarin in obese patients with AF due to their advantages of rapid onset of action, convenient dosing, and reduced drug interactions. Despite limitations in the study design and available data, our results are consistent with other meta-analyses and highlight the potential benefits of DOACs in this patient population. Further research is needed, particularly with regard to dabigatran, to confirm and expand upon these findings. Overall, the use of apixaban and rivaroxaban as anticoagulants in obese patients with AF appears to be effective in reducing major bleeding events, due to their predictable pharmacokinetic profiles. These findings have important implications for guiding clinical decision-making and improving anticoagulant therapy in obese patients with AF.

## References

[1] Okumura K, Yamashita T, Akao M (2020). Characteristics and anticoagulant treatment status of elderly non-valvular atrial fibrillation patients with a history of catheter ablation in Japan: subanalysis of the ANAFIE registry. J Cardiol.

[2] Bouame M, Ali Lahmar M, Bouafia MT (2018). Economic burden of thromboembolic and hemorrhagic complications in non-valvular atrial fibrillation in Algeria (the ELRAGFA study). J Med Econ.

[3] Kepez A, Erdoğan O (2013). Anticoagulation for non-valvular atrial fibrillation: new anticoagulant agents. Anadolu Kardiyol Derg.

[4] Hales CM, Carroll MD, Fryar CD, Ogden CL (2020). Prevalence of obesity and severe obesity among adults: United States, 2017-2018. NCHS Data Brief.

[5] Wang TJ, Parise H, Levy D, D'Agostino RB Sr, Wolf PA, Vasan RS, Benjamin EJ (2004). Obesity and the risk of new-onset atrial fibrillation. JAMA.

[6] Nalliah CJ, Sanders P, Kottkamp H, Kalman JM (2016). The role of obesity in atrial fibrillation. Eur Heart J.

[7] Tedrow UB, Conen D, Ridker PM (2010). The long- and short-term impact of elevated body mass index on the risk of new atrial fibrillation the WHS (women's health study). J Am Coll Cardiol.

[8] Lavie CJ, Pandey A, Lau DH, Alpert MA, Sanders P (2017). Obesity and atrial fibrillation prevalence, pathogenesis, and prognosis: effects of weight loss and exercise. J Am Coll Cardiol.

[9] January CT, Wann LS, Calkins H (2019). 2019 AHA/ACC/HRS focused update of the 2014 AHA/ACC/HRS guideline for the management of patients with atrial fibrillation: a report of the American College of Cardiology/American Heart Association Task Force on clinical practice guidelines and the Heart Rhythm Society in collaboration with the Society of Thoracic Surgeons. Circulation.

[10] Andrade JG, Verma A, Mitchell LB (2018). 2018 focused update of the Canadian Cardiovascular Society guidelines for the management of atrial fibrillation. Can J Cardiol.

[11] Wallace JL, Reaves AB, Tolley EA (2013). Comparison of initial warfarin response in obese patients versus non-obese patients. J Thromb Thrombolysis.

[12] Shaikh F, Wynne R, Castelino RL, Inglis SC, Ferguson C (2021). Effectiveness of direct oral anticoagulants in obese adults with atrial fibrillation: a systematic review of systematic reviews and meta-analysis. Front Cardiovasc Med.

[13] van Es N, Coppens M, Schulman S, Middeldorp S, Büller HR (2014). Direct oral anticoagulants compared with vitamin K antagonists for acute venous thromboembolism: evidence from phase 3 trials. Blood.

[14] Boonyawat K, Caron F, Li A (2017). Association of body weight with efficacy and safety outcomes in phase III randomized controlled trials of direct oral anticoagulants: a systematic review and meta-analysis. J Thromb Haemost.

[15] Hohnloser SH, Fudim M, Alexander JH (2019). Efficacy and safety of apixaban versus warfarin in patients with atrial fibrillation and extremes in body weight. Circulation.

[16] Rocca B, Fox KA, Ajjan RA (2018). Antithrombotic therapy and body mass: an expert position paper of the ESC Working Group on Thrombosis. Eur Heart J.

[17] Martin K, Beyer-Westendorf J, Davidson BL, Huisman MV, Sandset PM, Moll S (2016). Use of the direct oral anticoagulants in obese patients: guidance from the SSC of the ISTH. J Thromb Haemost.

[18] Alberts MJ, He J, Kharat A, Ashton V (2022). Effectiveness and safety of rivaroxaban versus warfarin among nonvalvular atrial fibrillation patients with obesity and polypharmacy. Am J Cardiovasc Drugs.

[19] Berger JS, Laliberté F, Kharat A (2021). Real-world effectiveness and safety of rivaroxaban versus warfarin among non-valvular atrial fibrillation patients with obesity in a US population. Curr Med Res Opin.

[20] Boivin-Proulx LA, Potter BJ, Dorais M, Perreault S (2022). Comparative effectiveness and safety of direct oral anticoagulants vs warfarin among obese patients with atrial fibrillation. CJC Open.

[21] Briasoulis A, Mentias A, Mazur A, Alvarez P, Leira EC, Vaughan Sarrazin MS (2021). Comparative effectiveness and safety of direct oral anticoagulants in obese patients with atrial fibrillation. Cardiovasc Drugs Ther.

[22] Chugh Y, Gupta K, Krishna HB, Ayala RQ, Zepeda I, Grushko M, Faillace RT (2023). Safety and efficacy of apixaban, dabigatran and rivaroxaban in obese and morbidly obese patients with heart failure and atrial fibrillation: a real-world analysis. Pacing Clin Electrophysiol.

[23] Costa OS, Beyer-Westendorf J, Ashton V, Milentijevic D, Moore KT, Bunz TJ, Coleman CI (2020). Rivaroxaban versus warfarin for management of obese African Americans with non-valvular atrial fibrillation or venous thromboembolism: a retrospective cohort analysis. Clin Appl Thromb Hemost.

[24] Deitelzweig S, Sah J, Kang A (2022). Effectiveness and safety of apixaban versus Warfarin in obese patients with nonvalvular atrial fibrillation enrolled in Medicare and Veteran Affairs. Am J Cardiol.

[25] Kido K, Ngorsuraches S (2019). Comparing the efficacy and safety of direct oral anticoagulants with warfarin in the morbidly obese population with atrial fibrillation. Ann Pharmacother.

[26] Kushnir M, Choi Y, Eisenberg R (2019). Efficacy and safety of direct oral factor Xa inhibitors compared with warfarin in patients with morbid obesity: a single-centre, retrospective analysis of chart data. Lancet Haematol.

[27] Perales IJ, San Agustin K, DeAngelo J, Campbell AM (2020). Rivaroxaban versus warfarin for stroke prevention and venous thromboembolism treatment in extreme obesity and high body weight. Ann Pharmacother.

[28] Peterson ED, Ashton V, Chen YW, Wu B, Spyropoulos AC (2019). Comparative effectiveness, safety, and costs of rivaroxaban and warfarin among morbidly obese patients with atrial fibrillation. Am Heart J.

[29] Weir MR, Chen YW, He J, Bookhart B, Campbell A, Ashton V (2021). Effectiveness and safety of rivaroxaban versus warfarin among nonvalvular atrial fibrillation patients with obesity and diabetes. J Diabetes Complications.

[30] Malik AH, Yandrapalli S, Aronow WS, Panza JA, Cooper HA (2019). Meta-analysis of direct-acting oral anticoagulants compared with warfarin in patients >75 years of age. Am J Cardiol.

[31] Malik AH, Yandrapalli S, Aronow WS, Panza JA, Cooper HA (2019). Oral anticoagulants in atrial fibrillation with valvular heart disease and bioprosthetic heart valves. Heart.

[32] Mhanna M, Beran A, Al-Abdouh A (2021). Direct oral anticoagulants versus Warfarin in morbidly obese patients with nonvalvular atrial fibrillation: a systematic review and meta-analysis. Am J Ther.

[33] Buck MM, Haddon AM, Paneccasio A (2021). Safety and efficacy of rivaroxaban and apixaban in patients with increased body mass: a systematic review. Clin Drug Investig.

[34] Jakowenko N, Nguyen S, Ruegger M, Dinh A, Salazar E, Donahue KR (2020). Apixaban and rivaroxaban anti-Xa level utilization and associated bleeding events within an academic health system. Thromb Res.

[35] Mocini D, Di Fusco SA, Mocini E (2021). Direct oral anticoagulants in patients with obesity and atrial fibrillation: position paper of Italian National Association of Hospital cardiologists (ANMCO). J Clin Med.

